# Brain-Derived Neurotrophic Factor (BDNF) as a Predictor of Treatment Response in Major Depressive Disorder (MDD): A Systematic Review

**DOI:** 10.3390/ijms241914810

**Published:** 2023-09-30

**Authors:** Mario Ignacio Zelada, Verónica Garrido, Andrés Liberona, Natalia Jones, Karen Zúñiga, Hernán Silva, Rodrigo R. Nieto

**Affiliations:** 1 Escuela de Medicina, Facultad de Medicina, Universidad de Chile, Santiago 8380453, Chile; 2 Clínica Psiquiátrica Universitaria, Hospital Clínico de la Universidad de Chile, Universidad de Chile, Santiago 8380453, Chile; 3 Departamento de Psiquiatría y Salud Mental Norte, Facultad de Medicina, Universidad de Chile, Santiago 8380453, Chile; 4 Departamento de Neurociencias, Facultad de Medicina, Universidad de Chile, Santiago 8380453, Chile

**Keywords:** neurotrophins, BDNF, biomarkers, treatment response, treatment resistance, treatment refractoriness, major depression, major depressive disorder, treatment-resistant depression

## Abstract

Brain-derived neurotrophic factor (BDNF) has been studied as a biomarker of major depressive disorder (MDD). Besides diagnostic biomarkers, clinically useful biomarkers can inform response to treatment. We aimed to review all studies that sought to relate BDNF baseline levels, or BDNF polymorphisms, with response to treatment in MDD. In order to achieve this, we performed a systematic review of studies that explored the relation of BDNF with both pharmacological and non-pharmacological treatment. Finally, we reviewed the evidence that relates peripheral levels of BDNF and BDNF polymorphisms with the development and management of treatment-resistant depression.

## 1. Introduction

Major depressive disorder (MDD) is currently understood as a systemic condition whose diagnosis is based on clinical findings. Several lines of research have been related to the complex and heterogeneous biological backgrounds of major depression, such as dysregulation of inflammatory responses, alterations in the hypothalamic–pituitary–adrenal (HPA) axis, as well as imbalances in neurotransmitter systems and neurotrophic factors, that together could contribute to the depressive symptoms observed in MDD [1].

One of these lines of research, considering neurotrophic factors, have studied the role of brain-derived neurotrophic factor (BDNF) in the neurobiology of major depression. Under physiological conditions, BDNF plays a role in promoting neuronal survival, in neurogenesis, neuronal differentiation and neuroplasticity. It has also been associated with the modulation of memory, learning and mood [2]. On the other hand, in animal models, it has been shown that exposure to stress reduces BDNF levels in the hippocampus and in cortical and subcortical regions [3]. In humans, changes in the expression of brain BDNF have also been described in response to certain disorders; lower levels of the factor have been identified in the anterior cingulate cortex and brainstem in suicidal subjects, as well as reduced adaptability to traumatic events among individuals with lower BDNF levels in specific regions of the brain [1].

Given the above, BDNF has been proposed as a potential MDD biomarker, postulating that the measurement of its peripheral levels, as well as the polymorphism and methylation status of the genes that encode it, could be used as predictors of disease severity or response to treatment or to evaluate the course of the illness [2]. Therefore, we aimed to review the current literature to study the evidence that supports the measurement of peripheral BDNF or the characterization of BDNF gene polymorphisms as biomarkers of treatment response in MDD patients.

## 2. Materials and Methods

We performed a systematic review of the literature available up to December 2022. In order to guarantee the quality of the articles, they were selected exclusively from indexed databases. The systematic search was carried out in the PUBMED database, and original research articles, meta-analyses, narrative and systematic reviews published in English or Spanish, until December 2022, were selected. The selected concepts were “BDNF” AND “Predicts” AND “Treatment” AND “Response”. From there, we selected those related to major depressive disorder. A second systematic search was carried out in the PUBMED database, and original research articles, meta-analyses, narrative and systematic reviews published in English or Spanish, until December 2022, were selected. The selected concepts were “BDNF” AND “Treatment” AND “Resistance” AND “Depression”.

The inclusion criteria were the following: empirical or primary studies, and reviews focused on the relationship between BNDF and response/resistance to depression treatment. The target population was human beings with no defined age limit, and quantitative, qualitative or mixed studies that were published in Spanish or English were included.

**Figure 1:**
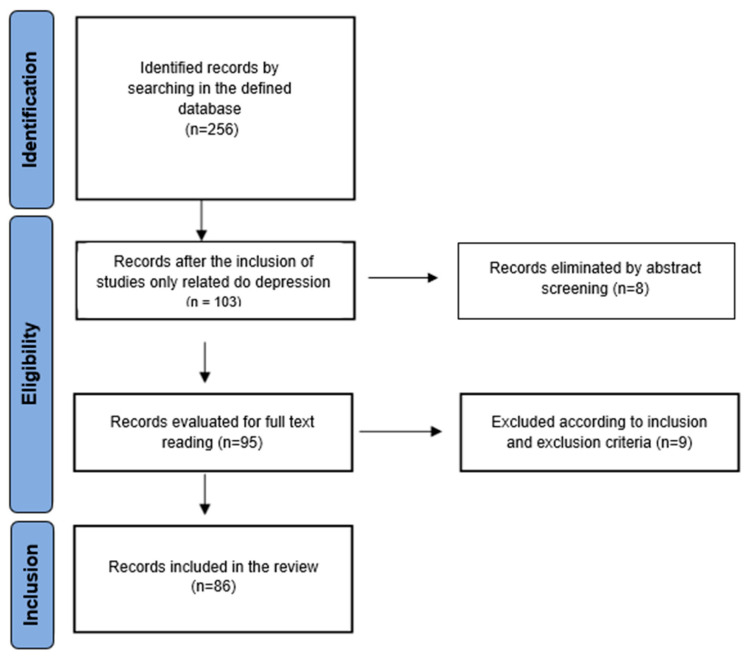
Flow chart of the literature search and study selection process in a systematic review of the literature on the relationship between BDNF and response to treatment of depression. Articles published up to December 2022.

**Figure 2:**
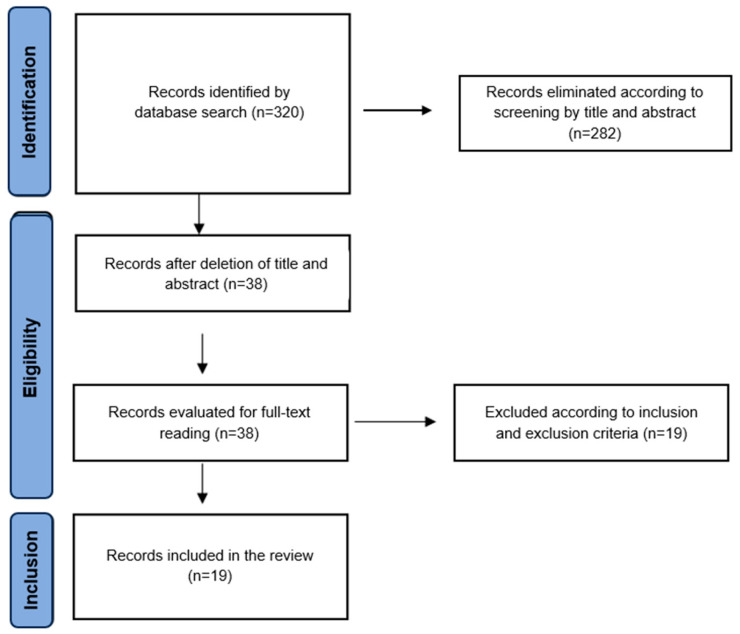
Flow chart of the literature search and study selection process in a systematic review of the literature on the relationship between BDNF and resistance to depression treatment. Articles published up to December 2022.

The PRISMA flow diagram was used to collect and filter the information. Initially, a total of 256 articles were obtained from the described database. After discarding in accordance with inclusion criterion 1 (studies focused on BDNF and depression), 103 studies were selected for abstract screening, with 95 studies being selected for full-text reading. For this purpose, all the studies were divided equally and randomly. Doubts and disagreements were resolved by discussion, and the final selection was made by consensus. Of the total, 81 studies were included in the systematic review (Figure 1). In relation to the second search, a total of 320 articles were initially obtained. Subsequently, they were screened by title, and 38 studies were selected for reading of the full text, with 19 studies being included in the systematic review (Figure 2).

## 3. Results and Discussion

### 3.1. BDNF Levels as a Biormarker of Treatment Response in MDD

Among the main lines of research on BDNF and its role as a biomarker, an attempt has been made to determine a correlation between its circulating levels and the diagnosis, severity and response to treatment in MDD, with an important group of publications supporting this association. A review of the literature published in 2018 highlights the role of BDNF as a biomarker given its ability to cross the blood–brain barrier and the correlation between its peripheral and central levels [4].

Regarding BDNF levels prior to the start of treatment, multiple studies have described lower levels of circulating factors in plasma compared with those in healthy individuals. A clinical study of 99 patients with MDD in 2022 described a significant reduction between their patients (40.26 ± 5.11 ng/mL) and healthy controls (47.21 ± 8.04 ng/mL) [5]. Another smaller study also showed lower BDNF levels in patients with MDD but found no correlation with the severity of depression. However, it was able to identify that higher levels of BDNF before treatment were associated with a better response to treatment [6]. A 2018 literature review also referred to lower basal BDNF levels in MDD, in addition to describing how neurotrophic factor deficiency was even more marked among geriatric patients [7]. On the other hand, a 2014 clinical study determined that higher basal levels of BDNF were associated with more severe MDD, but in turn, with better responses to treatment and early reductions in the Hamilton Depression Rating Scale (HAM-D) in the initial two weeks of treatment [8].

Multiple authors have identified that in response to effective treatment, BDNF levels tend to recover. A 2012 randomized controlled trial determined that a successful response was associated with increases in BDNF levels [9]. Another clinical study described that the lack of an early increase in serum BDNF, together with a lack of early improvement in symptoms, could predict future treatment failure with high specificity [10]. A recent review also described recoveries of BDNF levels in relation to effective treatments, which could imply that the neurotrophic factor could be at the end of a common pathway of the functioning of multiple of these treatments [7].

Despite the above, it is worth mentioning that other studies have not been able to reproduce these findings. Two longitudinal studies failed to determine correlations between pre-treatment BDNF levels and subsequent response in MDD and panic disorder [11,12]. One of these, carried out in 2015, also failed to identify correlations between BDNF levels and the severity of the symptoms according to the Montgomery Asberg Depression Rating Scale (MADRS) [12]. A clinical study evaluating 150 patients treated with Selective Serotonin Reuptake Inhibitors (SSRIs) also failed to correlate changes in serum BDNF levels in the first 4 weeks with respect to the end of treatment at week 8 [13]. Other authors studied subgroups of patients, such as those in whom MDD and obesity or a history of suicidality coexisted, without finding significant differences [2].

Some publications stated that although there is evidence of the predictive capacity of some biomarkers, such as BDNF, this was obtained from studies that were too small or had conflicting results, so that their practical use is still in doubt [14].

#### 3.1.1. BDNF Levels and Treatment Response to Specific Pharmacological Treatments in MDD

On the other hand, some studies have aimed to explore the role of BDNF as a biomarker of the response to certain pharmacological treatments.

A 2021 clinical study of 121 patients sought to determine the relationships between cognitive level in MDD, BDNF levels, and response to treatment with escitalopram and vortioxetine. Although both groups presented cognitive improvements in the tests carried out, BDNF demonstrated a predictive capacity in the case of vortioxetine, evidencing that higher basal levels were correlated with better verbal fluency and working memory [15].

A 2011 pilot study sought to determine the effect of venlafaxine treatment in 34 patients with MDD on serum BDNF levels. There was evidence of an increase in BDNF in the first two weeks of treatment among responders, while its levels were reduced in the sixth week of treatment among non-responders [16]. In 2015, an observational study compared clinical efficacy, safety and BDNF levels in patients treated with desvenlafaxine and fluoxetine, finding similar responses to treatment between both drugs and an increase in post-treatment BDNF levels in both groups of patients [17]. Other investigations focused on the study of SSRIs have determined that higher basal levels of BDNF prior to therapy are associated with better response to SSRIs, as well as an increase in the levels of the biomarker after the start of antidepressant treatment [18].

However, a study carried out in 2020 evaluated 83 adolescents aged 12–17 suffering from MDD, where it was shown that a successful eight-week treatment with escitalopram was associated with early reductions in plasma BDNF among responders (46 participants), compared with the non-responder group and the 56 controls. This contrasts with what was previously described in the literature. In this study, basal BDNF levels did not differ between controls and the MDD group, nor were they associated with MDD severity or future response to treatment [19].

There are other publications that have searched for the predictive characteristics of BDNF levels in MDD treatment. Two randomized studies carried out jointly in 2019 sought to identify the associations between response to treatment with paroxetine or venlafaxine and a series of biomarkers, among which BDNF levels were considered, where in the venlafaxine study, it was possible to demonstrate a positive correlation between BDNF levels and the intensity of the depressive symptoms, as well as an increase in BDNF levels as depressive symptoms decreased in women. Despite this, the differences were not significant [20]. A 2021 longitudinal study that comparatively evaluated the effect of vortioxetine with escitalopram, considering BDNF levels among its measurements, identified that treatment with the first antidepressant increases BDNF levels but found no correlation between its baseline levels and response to treatment. On the other hand, escitalopram is not associated with increases in BDNF nor do the baseline levels of the potential biomarker have an impact on the response to treatment [21].

In the same way, there are studies whose results concluded that there was no association between BDNF levels and the response to a certain pharmacological treatment. For example, in a 2017 study, few changes in serum/plasma BDNF levels were found to help predict the outcome of treatment with SSRIs or tDSC (non-invasive form of brain stimulation). Despite this, SSRIs are considered antidepressants that are associated with an increase in serum/plasma levels of BDNF in depressed patients; however, no differences were found in the baseline BDNF value between subjects with an outcome at positive and negative treatment; therefore, in this study, it was concluded that there are moderate predictive values in response to treatment [22].

A 2013 double-blind clinical study of 427 participants investigated the role of serum BDNF and other molecular biomarkers in response to desvenlafaxine treatment. That study concluded that no correlation was found between baseline biomarker levels and HAMD-17 severity and that treatment with desvenlafaxine 50 mg/d caused significant symptomatic improvement that did not correlate with biomarker measurements. Those changes from baseline were neither significant nor did they predict response to treatment at week 12 of the study. However, the study showed that the average change in BDNF was greater among patients with initially more severe depressions (HAMD-17 > 22) compared with less severe ones (33.4% vs. 4.3%) [23].

In 2019, a prospective cohort study was carried out with a sample of 267 older people diagnosed with depression using logistic regression (adjusted for covariates) with remission of depression after two years as the dependent variable and the baseline serum BDNF levels, childhood trauma and SSRI use as independent variables. The results showed that elevated BDNF levels were able to predict remission in patients traumatized in their childhood treated without SSRIs and in non-traumatized patients with SSRIs but not in other subgroups. It was concluded that early trauma may reduce the response of the neurotrophic system to SSRI treatment and whether this is of greater prognostic value than crude BDNF level is disputed [24].

#### 3.1.2. BDNF Levels and Treatment Response to Non-Pharmacological Treatments in MDD

Besides the lines of research of BDNF levels in relation to pharmacological treatments, the relationship between BDNF levels and other kinds of treatments for MDD has also been sought. In a 2019 randomized study, carried out in 22 patients with MDD, low-field magnetic stimulation (LFMS) was studied, being treated with rhythmic alpha stimulation (RAS) or rhythmic delta stimulation (RDS). The results showed that LFMS led to increases in BDNF levels during the 6 weeks of the study, with a substantial increase in RAS between weeks 4 and 6. It was also evidenced that the basal BDNF level was lower among responders than non-responders and increased more in responders than non-responders, with the change at week 2 being the most relevant in predicting response to treatment [25].

Other studies focused on the study of electroconvulsive therapy (ECT) presented results that suggest that there is no relationship between BDNF levels and the clinical outcome of this therapy. A 2007 study suggested that BDNF may not be associated with response to ECT overall in MDD but that some association may exist in subgroups, such as psychotic patients with MDD [26]. Similar findings were identified by a 2018 study, which concluded that ECT does not cause significant changes in serum BDNF but decreases plasma levels after the fifth session, concluding that there was no correlation with remission and the results were considered modest [27]. Similarly, in a 2019 observational study, it was shown that there is a certain relationship between BDNF and the response to ECT treatment in a late-onset depressive disorder; however, it is not considered sufficient and BDNF is ruled out as a useful marker [28]. In another prospective study from 2018, carried out in 20 patients with MDD, it was shown that the increase in peripheral BDNF levels is associated with the quality of the seizures during ECT; however, this increase was not significantly associated with an improvement in the HAM-D score [29]. In a 2021 review, ECT was found to increase neurotrophic factors such as BDNF and VEGF, but no association with clinical response was seen, and in terms of baseline levels, there was a correlation with VEGF but not with BDNF in response to ECT [30].

Regarding other treatments, a 2018 randomized study focused on investigating whether plasma levels of neurotrophic factors associated with major depression or antidepressant response predicted the effects of transcranial direct current stimulation (tDCS) concluded that BDNF fails to predict treatment response to tDCS in MDD [31]. Likewise, another study from 2022 focused on measuring the effectiveness of repetitive transcranial magnetic stimulation (rTMS) presented results where the basal levels of BDNF do not show differences between the control group and MDD, related neither to the severity nor to the level of cognitive deficit. In this study, only a marginal increase in BDNF levels was presented [32].

Other studied therapies were exercise and psychotherapy. For example, a 2020 controlled trial investigated 29 sedentary and depressed participants who were randomized to receive behavioral activation plus a prescription of exercise or stretching. The results showed that the patients who exercise present an increase in BDNF; however, there is no evidence that changes in BDNF are associated with an improvement in MDD [33].

Another controlled trial studied the role of BDNF and its interaction with working memory capacity in the results of psychotherapy for MDD. The results showed that elevated baseline levels of serum BDNF are associated with less depressive symptoms after psychotherapy, but only in the presence of high working memory capacity [34].

On the other hand, treatment with Ayahuasca, a serotonergic psychedelic, has been studied in a 2019 randomized controlled trial, determining that basal BDNF levels do not predict MDD and that after consumption of this substance, both patients and controls increase BDNF. Likewise, the results showed that 48 h after treatment, patients with MDD show an inverse correlation between depressive symptoms and high levels of BDNF. However, another randomized study from 2020 identified that there is no evidence of an association between BDNF and the response to treatment with Ayahuasca [35].

A post hoc analysis published in 2020 of a randomized double-blind study sought to evaluate the effects of a nutraceutical combination of omega-3, S-adenosyl-L-methionine, zinc, 5-hydroxytryptophan, folinic acid and a series of co-factors compared with a placebo in MDD, as well as the role of BDNF, among other biomarkers, as a predictor of response to this treatment; however, the neurotrophic factor did not show significant associations [36].

### 3.2. BDNF Polymorphisms as Biomarkers of Treatment Response in MDD

Several studies have focused on the BDNF gene, either studying its polymorphisms or its expression according to the methylation status of the BDNF gene, in relation to treatment response in MDD.

The role of BDNF polymorphisms as biomarkers of neuropsychiatric disorders, such as MDD, and as predictors of response to treatment has been studied in recent decades. Thus, a 2019 review indicates that the variation in a SNP (single nucleotide polymorphism) with the substitution of valine (Val) for methionine (Met) in codon 66 (Val66Met) has been related to neuropsychiatric disorders, adding that haploinsufficiency of the BDNF gene has been shown to give rise to profound adaptive behavioral dysfunctions, including affective symptoms in humans [37]. Regarding this same polymorphism, a 2017 review showed that BDNF Val66Met was associated with resistance to treatment with SSRIs and a possible relationship with lithium prophylaxis [38].

A 2022 study demonstrated that the Val66Met polymorphism was significantly associated with treatment response in depressed patients [39]. In another controlled study from the same year, it was highlighted that the Val66Met (rs6265), rs10501087 and rs11030104 polymorphisms is associated with treatment response in MDD [39]. In a 2021 study focused on analyzing the association of serum biomarker levels and BDNF gene polymorphisms with response to SSRIs, it was shown post-treatment that there was an increase in BDNF levels, in addition to a better response. It is associated with higher initial BDNF levels and the Val/Val polymorphism compared with Val/Met [18].

Similarly, in a review conducted in 2015, it was noted that in older adults with MDD, carriers of the Met66 allele were more likely to remit at 6 months. However, other studies in the same review found no association between this polymorphism and response to treatment [2]. Despite this, another study showed that serum BDNF was related to the response to mirtazapine in MDD, but the polymorphism was not. On the other hand, another study analyzed in the same review showed that the Val66Met polymorphism did not influence the response to mirtazapine but did lead to an increase in plasma BDNF [2].

In another 2011 study, a favorable association was found with the outcome of drug treatment for carriers of the Met/Met genotype [40]. However, another 2017 investigation indicated that there is a better therapeutic response to rTMS in patients with Val/Val homozygosity in the BDNF gene [41]. Likewise, in a 2016 review, it was pointed out that the rs6265 (Val66met) polymorphism is associated with lower secretion and mobility of intracellular BDNF, while on the other hand, the heterozygous variability (Val/Met) has better antidepressant efficacy. Similarly, it was mentioned that the Val/Val variant presents high levels of BDNF but low antidepressant efficacy and that the Met/Met variant is the one with the lowest levels and the worst response to antidepressants [42].

A 2020 review indicated that in older adult patients with MDD, there is a modest association with symptomatic improvement in the presence of BDNF rs6265 and greater remission in the presence of the Val/Met allele, suggesting that lower BDNF expression is associated with a better clinical expectation in this population over the Val/Val and Met/Met variants. Despite the above, there is greater evidence for other genes useful as biomarkers such as CYP2D6, SLC6A4 and 5-HTTLPR rather than for BDNF polymorphisms [43]. Regarding other polymorphisms, it has been seen that G196A (Val66Met/rs6265) and rs908867 influenced the outcome of antidepressant treatment, indicating that the BDNF G196A polymorphism partly determined a better antidepressant effect of both milnacipran and fluvoxamine [44]. In Chinese patients, the rs6265 polymorphism has been found to be associated with an early antidepressant response [2].

A 2010 study suggested that the BDNF gene may help predict the short-term response to citalopram and medication tolerability in MDD patients after TBI [45]. In a 2015 study, the response to SSRIs was analyzed, showing that patients with Val/Val polymorphism had a higher response rate 3 months after treatment compared with patients with Met. Similarly, in the same study, the response to serotonin and norepinephrine reuptake inhibitors (SNRIs) and tricyclics (TCAs) was studied, with patients with Val/Val having the lowest remission rate 6 months later compared with patients with Met. However, it should be noted that the sample size of this study was limited [46].

In another 2017 investigation, the relationship between childhood adversity and BDNF polymorphisms in the prediction of response to treatment with physical exercise and ICBT (Internet-based cognitive behavioral therapy) was studied. Met allele carriers without childhood adversity exposure or current antidepressant use were found to show a greater response to PE treatment than Val homozygotes. Similarly, there was no evidence to support that BDNF Val66Met or childhood adversity alone predicted response to PE and ICBT treatment. On the other hand, Met carriers had a higher serum mature BDNF level. These data suggest that carriers of the Met allele benefit most from physical exercise treatment, but only if they are not exposed to early adversity [47].

In a 2018 review, the findings suggest that increases in BDNF after ECT correlate with the effectiveness of seizures and treatment, with the CC genotype of the C270T polymorphism of BDNF being associated with the best response to treatment in patient’s psychotic-depressives and late-onset MDD, while other variations of C270T and G196A were unchanged. Despite this, it is mentioned that there is no clarity in the predictive role of BDNF in ECT, stressing the need to carry out new, more rigorous studies [48].

Another 2022 study focused on analyzing the relationship between BDNF polymorphisms, its serum levels, and its response to treatment with oral antidepressants and ECT concluded that the BDNF Val66Met polymorphism was associated with response to treatment with antidepressants in MDD, evidencing improvement in the group of patients with the Met allele. Likewise, there is evidence of a significantly higher plasmatic level of BDNF in MDD than in healthy individuals, and lower BDNF levels are associated with a better response to ECT in MDD [49].

In a 2018 international multicenter study, it was concluded that genotypic differences in BDNF Val66Met polymorphisms were associated with neither antidepressant effects [50] nor as predictors of response to active tDCS. Similar findings were found in a 2013 study aimed at studying the impact of the 5-HTTLPR and BDNF polymorphisms on the response to sertraline against transcranial direct current stimulation, evidencing that the BDNF polymorphism was not associated with the response to treatment, unlike the 5HTTLPR polymorphism [51].

Similarly, a study carried out in 2018 on 46 patients focused on evaluating whether the white matter integrity indices, the polymorphism linked to the serotonin transporter 5-HTTLPR and the BDNF Val66Met polymorphism had predictive capacity with respect to the magnitude of symptomatic change in depression after antidepressant treatment. The results showed the direct effect of the BDNF polymorphism on the neuronal connectivity of UF (uncinate fasciculus) and the moderating effect on the association between FA (fractional anisotropy, used as an indicator of white matter integrity) and the change in the severity of the depression. Thus, MDD patients with the BDNF Val/Val genotype exhibited higher FA values in the left UF relative to Met carriers. Second, depressed individuals who had elevated FA in the left UF and were Val/Val homozygotes exhibited greater improvements in depression severity after antidepressant treatment, in contrast to Met carriers. In addition, it was also evident that higher pretreatment FA values in the left UF predicted larger improvements in depression severity, with the Val66Met BDNF polymorphism moderating this association [52]. On the other hand, a 2020 meta-analysis concluded that this Val66Met polymorphism does not correlate with the effectiveness of antidepressants in MDD [53].

As previously described, there are various studies that analyze the relationship between BDNF polymorphisms with the presence or absence of response to treatment; however, other studies have concluded that there may be an even worse response in the presence of certain variants. For example, a 2010 investigation found that the BDNF rs7124442 TT phenotype was significantly associated with worse treatment outcome over 6 weeks in major depression, particularly anxious depression. In the same study, it was shown that BDNF rs7103411 and Val66Met rs6265 similarly predicted poorer response to treatment over 6 weeks in clinical subtypes of depression such as melancholic depression only. Despite this, it was concluded that the results do not support an association between genetic variation in BDNF and response or remission to antidepressant treatment [54]. Similarly, in a 2013 randomized study, it was identified that older adult patients who possessed the BDNF gene with the Met66 allele had a poorer response to antidepressant treatment with paroxetine [55].

On the other hand, in relation to methylation, a 2020 review indicated that patients with hypermethylation tend to receive more antidepressant treatments; however, they have shown that said methylation does not necessarily correlate with MDD severity. Likewise, in this same investigation, it was shown that hypomethylation of the promoter prior to treatment is associated with worse responses, while greater methylation of the BDNF promoter after treatment is associated with remission of depression [56]. This is consistent with what was evidenced in a 2018 study conducted in Chinese patients treated with escitalopram, in which the results suggest that BDNF DNA hypomethylation and its interaction with a lower SLE score (Life Events Scale) could cause a poor response to antidepressant treatment [57].

A 2018 review concluded that epigenetic modifications of the SLC6A4, BDNF and IL-11 genes are showing promising results as biomarkers for predicting antidepressant response. However, the research methods and results are heterogeneous and further studies are required before the results are generalizable [58]. On the other hand, in research related to modifications of specific positions, in a 2015 review, it was seen that the methylation status of position 87 of CpG could predict the response, evidencing that those patients who responded to the antidepressants increased methylation at the CpG 24 site and at the CpG 324 site [2].

Likewise, in a 2018 retrospective study in patients with MDD with severe depression and BDNF CPG-87 hypermethylation, it was shown that there was a better response to treatment in these patients compared with those with normal methylation. On the other hand, the same study showed that increases in methylation during the first 14 days of treatment in patients with symptomatic improvement are associated with greater specificity in predicting remission at the end of treatment, with the use of methylation achieving a similar effect of CpG-87 as a marker of early response [59].

A 2018 systematic review sought to address predictors of ketamine response in MDD, treatment-resistant depression (DRT) and bipolar affective disorder (BAD). Regarding the first, it was reported that the BDNF Val66Met SNP was associated with alterations in the activity of the same neurotrophic factor in the human brain, and it was believed that it could be related to the response to the drug, evidenced in a 2012 investigation in which the scores obtained with the HAM-D presented significantly smaller changes among those who carried Met compared with those who carried Val, which suggests a greater response to intravenous Ketamine among Val/Val BDNF carriers in rs6265e [60].

### 3.3. BDNF as a Biomarker of Response in Treatment-Resistant Depression (TRD)

Treatment-resistant depression (TRD) can be understood as a depressive disorder that, despite receiving two courses of indicated therapies under correct dosages and durations, fails to achieve a reduction of at least 25% in the severity of the symptoms [61]; however, there is significant variability between authors and consensus regarding this definition, its cut-off points and differentiation from other conditions such as partially responsive depression [61].

Due to the greater clinical challenge involved in developing a therapeutic scheme for these conditions, it has also been of interest to determine the role of BDNF as a predictor of the risk of developing TRD, as well as to evaluate possible therapeutic alternatives and to continuously evaluate the effectiveness of a given treatment when TRD is already established.

#### 3.3.1. BDNF Levels and Treatment Response to Pharmacological Treatments in TRD

There are several publications that describe the presence of reduced baseline levels of serum BDNF in patients who meet the TRD criteria [62].

In TRD, as in regular MDD, BDNF levels are influenced by ongoing antidepressant treatments. A 2019 clinical study carried out in Brazil that incorporated into its population a subgroup of 34 individuals with TRD identified a significant increase in circulating BDNF among them but stated that this finding was expected by its authors given that a significant percentage of them were receiving SSRI-based therapies; however, the authors stressed that there is still no clear consensus in the literature regarding increases in BDNF due to pharmacological therapies [63].

A 2019 meta-analysis found that peripheral measurements of total BDNF are inadequate predictors of treatment response in patients with TRD, as is individual measurement of its mature and precursor forms [64]. On the other hand, in a 2019 study that compared the production of BDNF in patients with TRD under conventional treatment, it was determined that although no differences were found in the gene expression, the plasmatic concentration of BDNF was increased in the TRD group, even with antidepressant treatment, so it could be used as a biomarker for the early identification of patients with TRD [63].

Interest in the study of BDNF as a marker of response to antidepressant treatments with ketamine has recently grown, given its mechanism of action; being an antagonist of the *N*-Methyl-D-Aspartic Acid (NMDAR) receptor with preferential binding to γ-aminobutyric acid-ergic (GABAergic) interneurons, this molecule induces both an increase in synaptogenesis and BDNF levels in interneurons [65,66]. A study of 15 adults with TRD evaluated the effects of ketamine infusions in sub-anesthetic doses over 4 weeks, evidencing a reduction in depressive symptoms quantified with MADRS, whose scores were found to be inversely correlated with the BDNF levels measured in plasma. In turn, basal BDNF levels predicted response to treatment in the first and fourth weeks [66].

Likewise, a 2014 randomized controlled trial aimed to determine the plasma BDNF levels in TRD and to assess their relationship with clinical outcomes. In parallel, they studied whether BDNF levels were predictors of the persistence of the antidepressant benefit of ketamine. The results of this trial show that ketamine increased the plasma BDNF levels of responders compared with those of non-responders. Similarly, plasma BDNF levels were negatively correlated with MADRS scores in patients receiving ketamine and were considered highly predictive of scores up to 72 h after ketamine infusion. These results support the hypothesis that early changes in plasma BDNF are associated with clinical outcomes for patients receiving ketamine therapy for TRD [67]. Other reviews have reached results similar to the above, being the case of an article published in 2020 that reported that in TRD, a positive response to ketamine therapy is associated with early increases in the measurement of circulating BDNF. In turn, it describes that higher levels of the biomarker are correlated with clinical improvement when using this drug [68].

Another investigation that studied the relationship between BDNF levels with the response to ketamine was carried out in 2013, this time evaluating it in relation to a single dose of the drug. The results show that between ketamine responders and non-responders, there are no differences in baseline BDNF. However, non-responders 7 days after starting treatment presented reductions in circulating BDNF levels. Similarly, increases in BDNF were observed among responders, but with high variability and without statistical significance [69].

Other studies have focused on making comparative relationships between different treatments in patients with TRD. This is the case of a study carried out in 2015 in 35 patients who received up to 12 ECT sessions or up to 3 ketamine infusions, in which it was found that serum BDNF was lower in patients with TRD at the beginning of the study compared with that of healthy controls. In addition, it was found that treatment with ketamine and ECT was associated with significant reductions in depressive symptoms. However, serum BDNF rose significantly only one week after the first ketamine infusion in responders, but not in subsequent infusions. Therefore, the study concludes that serum BDNF normalization does not seem to be a prerequisite for symptomatic improvement in TRD after treatment with ketamine or ECT [70].

On the other hand, studies have been carried out in relation to the complementation of antidepressant treatment with antipsychotic drugs. This is how, in 2010, the effects of several atypical antipsychotics as adjuvants to antidepressants were examined, evidencing that among the responders who reduced their HAM-D scores by >50%, there was a significant increase in BDNF up to week 4 of observation, which in turn correlates with the level of improvement [71].

Within this same line of study, in a 2008 investigation carried out in 25 patients (4 men and 21 women) from 27 to 67 years of age with bipolar depression who received mood stabilizing drugs, it was determined that the addition of risperidone to sertraline is effective and well tolerated by depressive patients resistant to the latter, which is accompanied by increased serum BDNF levels in those who respond to the addition of risperidone, also determining that the addition of risperidone does not influence the metabolism of sertraline [72].

#### 3.3.2. BDNF Levels and Treatment Response to Non-Pharmacological Treatments in TRD

Regarding the use of ECT in TRD, the vast majority of recent studies agree with a lack of correlation with BDNF levels, at least in the context of TRD. A clinical study of 74 participants published in 2019 sought to evaluate the relationship between the BDNF genotype and its peripheral levels against the application of ECT. Measurements were made prior to therapy, the day after it and 1 month later; however, no relationships were identified between biomarker levels and the degree of immediate response, sustained response or remission against the disease [64]. Another study, previously published in 2015, also sought to determine the impact of ECT on circulating sBDNF levels. The authors reported having identified lower basal levels of sBDNF among patients with TRD compared with healthy controls; however, these levels were not altered after the application of ECT nor were they related to the degree of symptomatic improvement resulting from therapy [70]. Another smaller study from 2020 also agreed with the findings of previous research [63], as did another earlier study from 2013 that also failed to identify associations between BDNF and HAMD-17-measured symptomatic improvement [73]. A 2015 study that subjected 21 TRD patients to ECT also failed to identify significant correlations [74].

Only three studies, two older, described ECT influences on BDNF levels. A 2006 study with 23 patients showed an increase in peripheral BDNF after 1 month of treatment application. In addition, the author added that although BDNF did not change in the majority of patients immediately after ECT was performed, it was possible to see early increases among the patients who had significantly reduced BDNF levels prior to the start of therapy [75]. In turn, a review of the literature carried out in 2009 regarding ECT and its generalities suggested that among its studied publications, increases in BDNF have been described in patients with TRD who respond to ECT, citing a 2008 study that referred to an increase from 8 ng/mL to 15 ng/mL in peripheral BDNF at the fifth week after treatment [76].

The third study supporting the predictive role of BDNF in ECT, published in 2022 and conducted in a cohort of 23 patients, identified that future remitters showed significantly higher levels of mBDNF (mature BDNF, a BDNF isoform involved in the neuronal plasticity and the survival of neural networks). The age- and sex-controlled multiple logistic regression model used in the study revealed that having a higher baseline mBDNF level was significantly associated with future remission after ECT sessions [77].

Regarding rTMS, a recent 2021 study sought to identify its effects on serum BDNF as well as VEGF and TNFa in TRD and schizophrenia. Of the 25 TRD patients enrolled, rTMS achieved an average 30% reduction in MADRS and HAM-D tests. Throughout the three measurements, it was possible to show a successive increase in BDNF, with a statistically significant change between the first (26.37 ng/mL) and third (29.69 ng/mL) measurements according to the related samples *t* test (t = −2.601; *p* = 0.016). During treatment, the study was able to show an inverse correlation between the percentage increase in BDNF and the severity of the clinical pictures according to MADRS and HAM-D but failed to identify similar correlations after treatment. Due to the latter, the authors concluded that the measurement of BDNF did not have significant correlations with respect to the outcome of the treatment. However, the authors observed that higher initial MA-DRS and HAM-D scores were associated with lower increases in BDNF during treatment [78].

Similarly, in a 2014 study conducted in 19 women with TRD, the authors analyzed the individual effects of rTMS treatment in association with polymorphism in the BDNF gene and found a slightly better, but not significant, response for a group of Val66Val homozygotes [79]. A double-blind study of 22 patients with TRD carried out in 2013 sought to evaluate the effect of tDCS in relation to serum BDNF levels. The clinical response described by the authors was reduced, with a moderate improvement in the clinical tests performed without statistical significance. Serum BDNF levels were measured at baseline, at 2 weeks and at 4 weeks, but no significant changes or association with the symptoms were identified [80].

#### 3.3.3. BDNF Polymorphisms and Treatment Response in TRD

As in MDD, the TRD literature has also addressed the role of the various BDNF polymorphisms. On the one hand, it has been shown that in TRD, the genetic expression of BDNF can be altered in a similar way to that described in MDD. A 2014 study was conducted on 50 patients (26 with TRD and 24 with treatment-responsive MDD) enrolled for BDNF mRNA measurement. As expected, mRNA levels were both decreased in MDD and TRD patients compared with those of healthy controls; however, the study determined that this decrease was more significant among TRD patients, even without significant differences in HAM-D scores between both groups, so the authors did not attribute the difference to the severity of the symptoms but to more severe neurotrophic dysfunctions in TRD [81].

A 2012 prospective longitudinal multicenter study in 948 Han patients sought to determine the risk conferred by multiple polymorphisms, including BDNF and NTRK2, in relation to the risk of developing TRD in patients with MDD in course. Regarding BDNF (rs6265), it failed to determine statistically significant differences between the studied groups, although it did determine that the rs1565445 polymorphism of the NTRK2 gene was associated with a greater risk of TRD. The publication states that although the importance of the Val66Met polymorphism in BDNF (rs6265) has been described in the literature, these findings have been difficult to reproduce in the Asian population such as the one present in the study, so the authors conclude that this BDNF variation may not play a major role in the Han-Chinese population. On the other hand, after carrying out a cross combination between the different studied genotypes and polymorphisms, a lower, but significant, interaction was found between BDNF (rs6265) and NTRK2 (rs1387923, rs2769605 and rs1565445), possibly due to gene–gene interaction phenomena regarding the development of resistant MDD [82].

In a study carried out in 2019 by the European Group for the Study of Resistant Depression (GSRD), it was found that the application of machine learning algorithms, together with the presence of three SNPs of the BDNF, PPP3CC and HTR2A genes and lack of melancholy, predicted response to treatment. Thus, that study indicated that the literature describes the Val66Met functional polymorphisms in BDNF—rs11030104 and rs11030101—as indicators of less effective treatment without affecting suicidality, while the machine learning algorithm used in that study identified BDNF SNP GG of rs6265, together with the SNPs of HTR2A and PPP3CC in the absence of melancholy, as frequent variations in response to effective treatments [83].

On the other hand, the effect that these genetic variations have on the effectiveness of certain pharmacological treatments has been investigated. A recent 2021 study sought to perform a genome-wide association study (GWAS) regarding response to low-dose Ketamine infusion treatment. From the genotyping of 64 patients, 12 genes involved in the response to the drug were identified, where SNPs and complete genes related to BDNF-TrkB signaling pathways stood out as key players in the response to its pharmacological effect, highlighting rs2049048 in BDNF and rs10217777 in its receiver, NTRK2. It was evidenced that the presence of these SNPs, together with others of the glutamatergic and GABAergic pathways, were associated with rapid antidepressant responses in a range of 240 min with prolonged durations of up to 2 weeks [84].

Regarding ECT, the previously mentioned study by Maffioletti et al., (2019), in addition to seeking to evaluate the response of BDNF levels against ECT, also carried out an analysis regarding the impact of the Val66Met polymorphism on therapy, but like their conclusions regarding biomarker levels, they did not identify a role for the polymorphism either, due to overresponse to treatment and remission with this therapeutic strategy [64].

A 2013 study conducted on a cohort of 119 patients with TRD evaluated the efficacy of ECT in terms of its ability to reduce MADRS scores in relation to the presence of BDNF polymorphisms. It was determined that the rs11030101 polymorphism was associated with changes in the scale, evidencing a lower possibility of benefit from ECT in the presence of the TA genotype compared with that of the TT genotype. rs61888800 was also studied, but no significant association [85] was attributed to it.

Regarding rTMS, a 2008 clinical study sought to determine the role of the 5-HTTLPR polymorphism of the gene encoding the serotonin transporter (SLC6A4) and Val66Met in BDNF. The study was carried out in a group of 36 patients with TRD who, maintaining their previous pharmacological therapies, underwent five consecutive morning sessions of rTMS. In turn, a double-blind study was carried out in parallel with the same group of patients, subjecting a subgroup of these to a placebo treatment. Treatment with rTMS significantly improved depression symptoms (*p* < 0.0001), and the response was significantly higher in 5-HTTLPR LL homozygotes compared with S allele carriers (*p* = 0.007) and in homozygous BDNF Val/Val cough compared with carriers of the Met allele (*p* = 0.024). No significant influence of these polymorphisms was observed in terms of clinical improvement in the placebo group. Despite this, it is emphasized that more research and larger samples are needed to clarify the utility of 5-HTTLPR and BDNF Val66Met genotyping in optimizing non-pharmacological treatments in mood disorders [86]. Likewise, a 2015 review, whose objective was to systematically synthesize the literature on the neurobiological predictors of rTMS in patients with depression, determined that the response to treatment can be predicted with the basal blood flow of the frontal lobe using the presence of gene polymorphisms: 5-hydroxytryptamine (5-HT)-1a, the 5-HTTLPR genotype LL and BDNF Val/Val homozygotes [87].

## 4. Discussion

MDD is a neuropsychiatric disorder ranging from mild to severe, affecting millions of people worldwide and causing significant losses every year in terms of the quality of life for those who suffer from it. It also has major negative economic impacts on healthcare systems. The treatment of MDD varies widely from patient to patient, and sometimes, it takes longer than a year to find the best treatment approach for an individual patient. In this regard, there is a need in psychiatry to identify well-studied factors that can predict treatment responses at different stages of the disease and with various pharmacological and non-pharmacological treatment options.

In this article, we attempted to address the question of whether BDNF serum levels or polymorphisms are predictors of treatment response in patients suffering from MDD. Despite inconsistent results, the analyzed studies tended to indicate that BDNF serum levels are lower in patients with MDD compared with those in healthy controls, and pharmacological treatments usually lead to an increase in these levels, which correlates with an improvement in the clinical condition. The association is less clear when analyzing non-pharmacological treatments, as there is more controversy in the literature.

Regarding BDNF polymorphisms, Val66Met appears to be the most related to treatment response, but a definitive conclusion cannot be reached due to the heterogeneity of the considered studies. However, it can be said that BDNF polymorphisms play a role in the pathogenesis and response that need further characterization and definition.

Now, considering TRD, there is also some evidence supporting BDNF as a biomarker for treatment response in pharmacological approaches, especially with ketamine. However, there is some consensus that it is not a good predictor of response for treatments other than pharmacological ones.

Comparing the findings of the present study with other relevant publications, it can be highlighted that even though BDNF and its polymorphisms could hold a role as predictors of treatment response, the studies behind said statement have a tendency to be contradictory and inconsistent.

Cavaleri highlighted that subjects suffering from MDD have lower central and peripheral BDNF levels than non-depressed individuals [88], a finding that coincides with our search where lower levels of the circulating factor in plasma prior to treatment initiation have been described, compared with those in healthy individuals [5]. On the other hand, Cavaleri observed an increase in peripheral BDNF levels in relation to symptom improvement along with an inverse correlation between peripheral BDNF and symptom severity [88], results that are concordant with the studies in our review [6,10,12]. On the other hand, in Kishi, 2018, decreased baseline levels of BDNF were identified in patients who were depressed, but they were reported to be not specific to MDD. Nevertheless, the author did identify a correlation between the increase in peripheral BDNF levels and treatment response [89]. Similarly, it was noted that there were no associations between BDNF levels and suicidal tendencies both by Cavaleri and within our results [1,2,85].

Within Cavaleri’s search, it was noted that people without antidepressants tend to have lower BDNF concentrations than people receiving some form of treatment. However, it was stated that this finding was inconsistent and subject to a significant risk of publication bias [88]. Similarly, significant increases in BDNF were found in relation to the use of SSRIs and SNRIs, which is consistent with our findings; on the other hand, Kishi (2018) also reported similar findings in relation to SSRIs, but they did not find a significant correlation with SNRIs [89]. However, for Cavaleri, data on other types of antidepressants and studies focused on a single type of antidepressant agent were limited, although he described a significant increase in BDNF with sertraline and non-statistically significant changes with paroxetine, escitalopram and venlafaxine [88]. Within our results, one study reported that the use of escitalopram is associated with early reductions in plasma BDNF among responders [19], which is not consistent with those reported by Cavaleri.

It is noted that in Cavaleri’s publication, there were no significant variations in BDNF levels in relation to ECT, rTMS and physical activity [88]. Kishi showed similar findings; however, they highlighted an increase in BDNF levels in ECT, even though it had no correlation with treatment response [89]. This coincides with our results in which it was found that there is no significant relationship between BDNF and the response to these therapies.

In relation to the Val66Met polymorphism, Kishi (2018) suggested that this genetic variation was not associated with an increase risk of MDD in an European cohort, even though the authors reported that this may be due to the limited number of studies on the subject, but they did identify an increased risk of developing late-life MDD in relation to the polymorphism. However, they referred to an association between Val66Met and a response to antidepressant treatment [89]. In our review, we found contradictory findings regarding this polymorphism and treatment response; some studies suggested that its presence was related to resistance to treatment response, such as with SSRIs [38], a finding that is also consistent with the results of another study that shows a better response to SSRI treatment with Val/Val over Val/Met [47]. Other studies have also described Val/Val as a positive marker for treatment response over Val66Met [52]. Even though some studies support the notion that Val/Met could indeed be linked to better antidepressant efficacy [43,50], it seems likely that this is highly dependent on the characteristics of the cohort in question, since other studies only describe a positive association between Val66Met and clinical outcomes in specific populations, such as older adults, on Chinese cohorts and in individuals undergoing treatment with milnacipran or fluvoxamine [2].

In terms of limitations presented in our study and in the literature that was analyzed, a risk of bias was identified due to study heterogeneity, which was also referred to by Cavaleri. Heterogeneity in the characteristics of the sample (such as diagnosis tools; DSM vs. ICD; and used methods, for example BDNF measurements methods and timing of the measurements) could have induced heterogeneity in the results and therefore in the conclusions on the matter.

It must be noted that the studies that were incorporated in this review used peripheral levels of BDNF as an indirect measurement of central BDNF levels, a practice that is supported by several studies that suggest a good correlation between peripheral and central levels of said neurotrophic factor. Despite that, it has been described that BDNF can also be peripherally produced by non-neural tissue and is stored and released by platelets during clotting; therefore, seric levels of BDNF may differ from plasma levels of BDNF, which is the reason why serum BDNF is usually preferred for this type of study. Despite that, there were studies that did not differentiate between them or used them interchangeably, which may have affected their results [90].

Furthermore, some of the analyzed studies did not account for some characteristics of their samples such as subject age, age of diagnosis of MDD, number of depressive episodes, bodyweight or lifestyle factors (alcohol consumption, smoking and diet), which where also described in a non-insignificant number of publications included in this review as confounding factors and limitations for their findings.

Also, most of the reviewed studies that analyzed the association between BDNF levels and treatment response did not account for the potential differences between never-previously medicated patients with MDD and those who had undergone treatment in the past.

It must also be taken into account that not all present studies have explicitly reported their measurement methods for BDNF. This is of relevance since prior to high sensitivity kits for BDNF and proBDNF, specificity was limited since earlier versions of these kits were unable to differentiate between proBDNF and mature BDNF. [91] This is of relevancy because both proBDNF and mature BDNF are active, showing opposing effects via the p75NTR and tropomyosin-related kinase B (TrkB) receptor, respectively, for example, as seen in studies that have identified a decrease in the production of mature BDNF and an increase in the levels of pro-peptide BDNF in the parietal cortex of MDD, schizophrenia and bipolar disorder groups in comparison with controls [92].

Finally, our methodological approach to this review limited our sources to a single database; thus, we cannot exclude the risk of leaving out studies on other databases that could have met our inclusion criteria.

## 5. Conclusions

Given the current prevalence of MDD worldwide and the relevance of its impact on both quality of life and economic level, it is of utmost importance to develop studies focused on improving both diagnosis and treatment, as well as follow-up markers in relation to the response of the latter.

In this regard, BDNF has been studied as a marker of possible response to treatment, and a decrease in BDNF levels in MDD has been described, determining that pharmacological treatments could increase BDNF levels associated with clinical improvement. The expectation of a better response in patients with higher baseline levels is something that still needs more evidence. However, early increases in BDNF levels after treatment initiation could be an indicator of a good response. Of note, findings with non-drug therapies have not been clear or significant.

On the other hand, BDNF polymorphisms have also been the focus of research; however, given the diversity among study orientations, there are no concrete results either.

In conjunction with the above, the variability between studies in relation to the age of the patients, the type of diagnosis, the different diagnostic tools used, the age of onset of MDD, the number of depressive episodes, the dose and duration of therapy, different samples with a history of previous treatment vs. virgin patients to treatment and the different methods of measuring BDNF are some of the limitations that we face in the analysis of our results.

Lastly, it is worth mentioning that MDD being a systemic condition encompasses not only the highly complex biomolecular and neurophysiological pathways described in this review but also the psychosocial ones that are maintained within the social sphere, healthcare system and environment where an individual is situated. Thus, a patient’s response to treatment cannot depend solely on the levels of a biomarker such as BDNF and its polymorphisms; instead, they should be incorporated into a larger MDD scoring system that could integrate an individual’s biological, ethnic, social and psychological characteristics, offering together a much more accurate prognosis predictor for potential therapeutic approaches.

We consider that a study that manages to homogenize, to a large extent, the variables mentioned above would be of great relevance both for the theoretical study of BDNF as a marker of response to treatment and for its subsequent application in clinical practice. Another approach that we consider valuable is to delve further into non-pharmacological therapy since their results within the current literature are not entirely conclusive, as well as studies in relation to BDNF polymorphisms. We hope that, in the future, the accumulation of evidence of responses to different treatments in patients with different genetic backgrounds may help clinicians to choose a personalized treatment for each patient.

## Data Availability

The data used for the preparation of this manuscript can be found in PubMed, a publicly available database.
